# Changes in global histone modifications during dedifferentiation in newt lens regeneration

**Published:** 2010-09-16

**Authors:** Nobuyasu Maki, Panagiotis A. Tsonis, Kiyokazu Agata

**Affiliations:** 1 Department of Biology and Center for Tissue Regeneration and Engineering at Dayton, University of Dayton, Dayton, OH; 2 Center for Developmental Biology, RIKEN Kobe, Kobe, Japan; 3 Department of Biophysics, Graduate School of Science, Kyoto University, Kyoto, Japan

## Abstract

**Purpose:**

Reprogramming of pigmented epithelial cells (PECs) is a decisive process in newt lens regeneration. After lens removal PECs in dorsal iris dedifferentiate and revert to stem cell-like cells, and transdifferentiate into lens cells. Our purpose is to know how global histone modifications are regulated in the reprogramming of PECs.

**Methods:**

Iris sections were stained using various histone modification-specific antibodies. The intensity of stained signal in nucleus of PECs was measured and changes in histone modification during dedifferentiation were evaluated.

**Results:**

During dedifferentiation of PECs histone modifications related to gene activation were differentially regulated. Although tri-methylated histone H3 lysine 4 (*TriMeH3K4*) and acetylated histone H4 (*AcH4*) were increased, acetylated histone H3 lysine 9 (*AcH3K9*) was decreased during dedifferentiation. Among all gene repression-related modifications analyzed only tri-methylated histone H3 lysine 27 (*TriMeH3K27*) showed a significant change. Although in the dorsal iris *TriMeH3K27* was kept at same levels after lentectomy, in ventral iris it was increased.

**Conclusions:**

Histone modifications are dynamically changed during dedifferentiation of PECs. A coordination of gene activation-related modifications, increasing of *TriMeH3K4* and *AcH4* and decreasing of *AcH3K9*, as well as regulation of *TriMeH3K27*, could be a hallmark of chromatin regulation during newt dedifferentiation.

## Introduction

Newts have impressive regenerative capabilities ranging from tissue repair to replacement of whole organs. Their lens regenerative capability in adults is a unique feature of newts when compared to other regenerative animals such as axolotl, frog [1] and zebrafish [unpublished data]. In newt lens regeneration lens is regenerated by transdifferentiation of pigmented epithelial cells (PECs). After lens removal PECs from the dorsal iris start dedifferentiating, during which they shed off their pigment granules and re-enter the cell cycle. The dedifferentiated cells continue to proliferate and finally differentiate into lens cells. This transdifferentiation has been also demonstrated by clonal culture experiments [2]. Recently it was shown that nucleostemin, a nucleolar protein mainly expressed in stem cells, and three of the four factors necessary to induced pluripotent stem cells, cellular myelocytomatosis (c-Myc), Kruppel-like factor 4 (Klf4), and sex determining region Y-box2 (Sox2), are expressed in the dedifferentiated PECs [3,4]. This suggests that PECs reprogram to a stem cell-like state during dedifferentiation.

In contrast to dorsal iris, ventral iris does not contribute to lens regeneration even though the shedding of pigment granules and cell cycle reentry occurs in ventral PECs as well. It has been shown that inhibition of bone morphogenetic protein (BMP) signaling [5] and wingless and integration (Wnt) signaling [6] in dorsal iris after dedifferentiation could be important to determine lens regeneration from dorsal iris. However, the mechanism that mediates a dorso-ventral identity at earlier stage has not been clarified.

Covalent modifications in histone tails are epigenetic events that regulate a wide variety of biological processes during cellular differentiation and development [7]. To know whether histone modifications are involved in dedifferentiation of PECs and dorso-ventral selectivity of lens formation, changes in global histone modifications were analyzed.

## Methods

### Animals

Japanese common newts, *Cynops pyrrhogaster*, were collected in the northern part of Okayama prefecture, Japan. For this study, male newts (6.0±0.5 g) were used. All animal procedures were approved by animal care board in Center for Developmental Biology, Riken Kobe. Newts were euthanized by anesthesia (soaking in 0.1% of MS-222; Sigma-Aldrich, Tokyo, Japan, for 15 min) followed by decapitation.

### 5-bromo-2’-deoxyuridine administration

To examine cells re-entering to cell cycle, 5-bromo-2’-deoxyuridine (25 mg/g bodyweight) was intraperitoneally administrated everyday from one day before lentectomy.

### Immunohistochemistry

Eye balls were collected and fixed in methanol-acetic acid (3:1) at 4 °C for 12 h and embedded in paraffin. Paraffin sections (20 µm) were deparafinized and incubated with 2× SSC with 0.05% Triton X-100 and 0.05% saponin (MP Biomedicals Japan, Tokyo, Japan) for 60 min. The sections were rinsed with 2× SSC for 15 min 3 times, blocked in TNB buffer supplied with the TSA kit (Perkin Elmer, Waltham, MA) for 60 min, and incubated with each antibody against histone modification and anti-BrdU mouse monoclonal antibody (MAB3510; Millipore, Billerica, MA) at 4 °C overnight followed by Alexa-488 conjugated anti-mouse IgG antibody (Invitrogen, Carlsbad, CA) and Cy3-conjugated anti-rabbit IgG antibody (Millipore) at room temperature for 90 min. The nuclei in the sections were counterstained with Hoechst 33258. Antibodies against histone modifications, TriMeH3K4 (ab8580; Abcam, Cambridge, UK), AcH3K9 (ab10812; Abcam), di-methylated histone H3 lysine 9 (DiMeH3K9, 07–212; Millipore), TriMeH3K9 (ab8898; Abcam), DiMeH3K27 (ab24684; Abcam), and TriMeH3K27 (07–449; Millipore), and AcH4 (06–598; Millipore), were used. These antibodies are known to interact with modified histones in a variety of animals ranging from yeast to humans.

### Image acquisition and signal measurement

The images were taken on a Axiovert 200M microscope (Zeiss Japan, Tokyo, Japan) using a 512BFT CCD camera (Nippon Roper Scientific, Tokyo, Japan). To measure the signal intensities taken images were saved in TIFF format. The average signal intensities per pixel of histone modifications and Hoechst 33258 in each nucleus were measured using MetaMorph software (ver. 7; Molecular Devices, Sunnyvale, CA) without any image processing. The intensity of each histone modification was normalized with that of Hoechst 33528.

## Results and discussion

**Figure 1:**
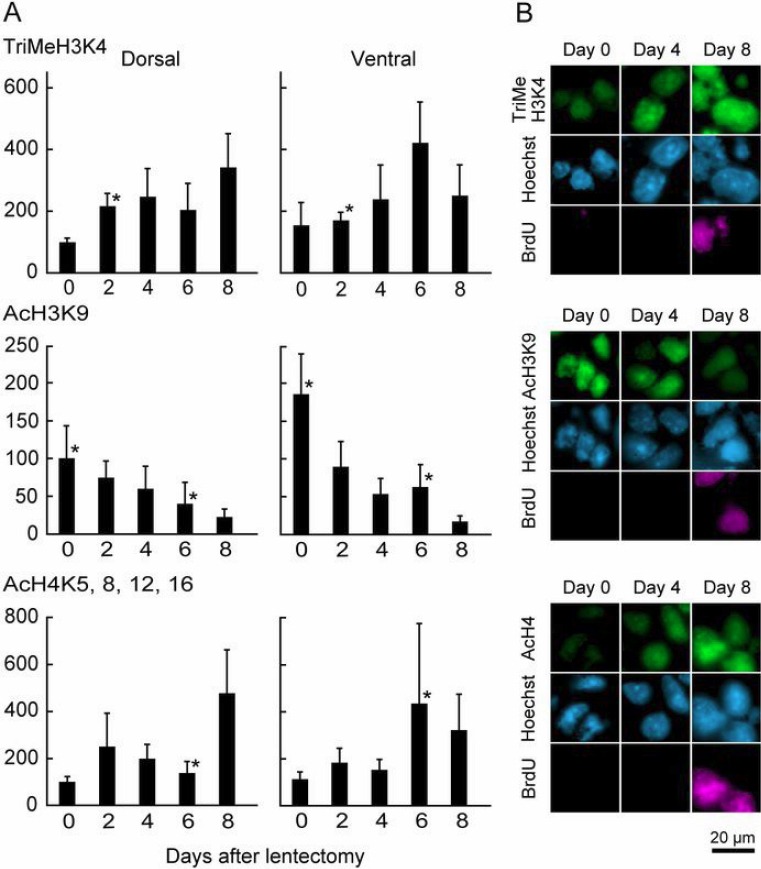
Changes in histone modifications related to gene activation during early lens regeneration. **A**: Quantification of detected signal by immunohistochemistry using histone modification antibodies. To examine the cells before cell cycle reentry, BrdU was administrated everyday. Immunohistochemistry was performed and the detected signal intensity of each histone modification in nucleus of PECs without BrdU incorporation was measured. The signal intensity of histone modification was normalized with that of Hoechst 33528. The value of normalized signal in dorsal iris on day 0 is represented as 100%. Error bars, standard deviation (n=5–17). Asterisks indicate a significant difference at p<0.03, Student’s *t* test (2-tailed) between dorsal and ventral iris at same day. **B**: Immunohistochemistry. Staining patterns in dorsal iris are shown at different time points.

**Figure 2:**
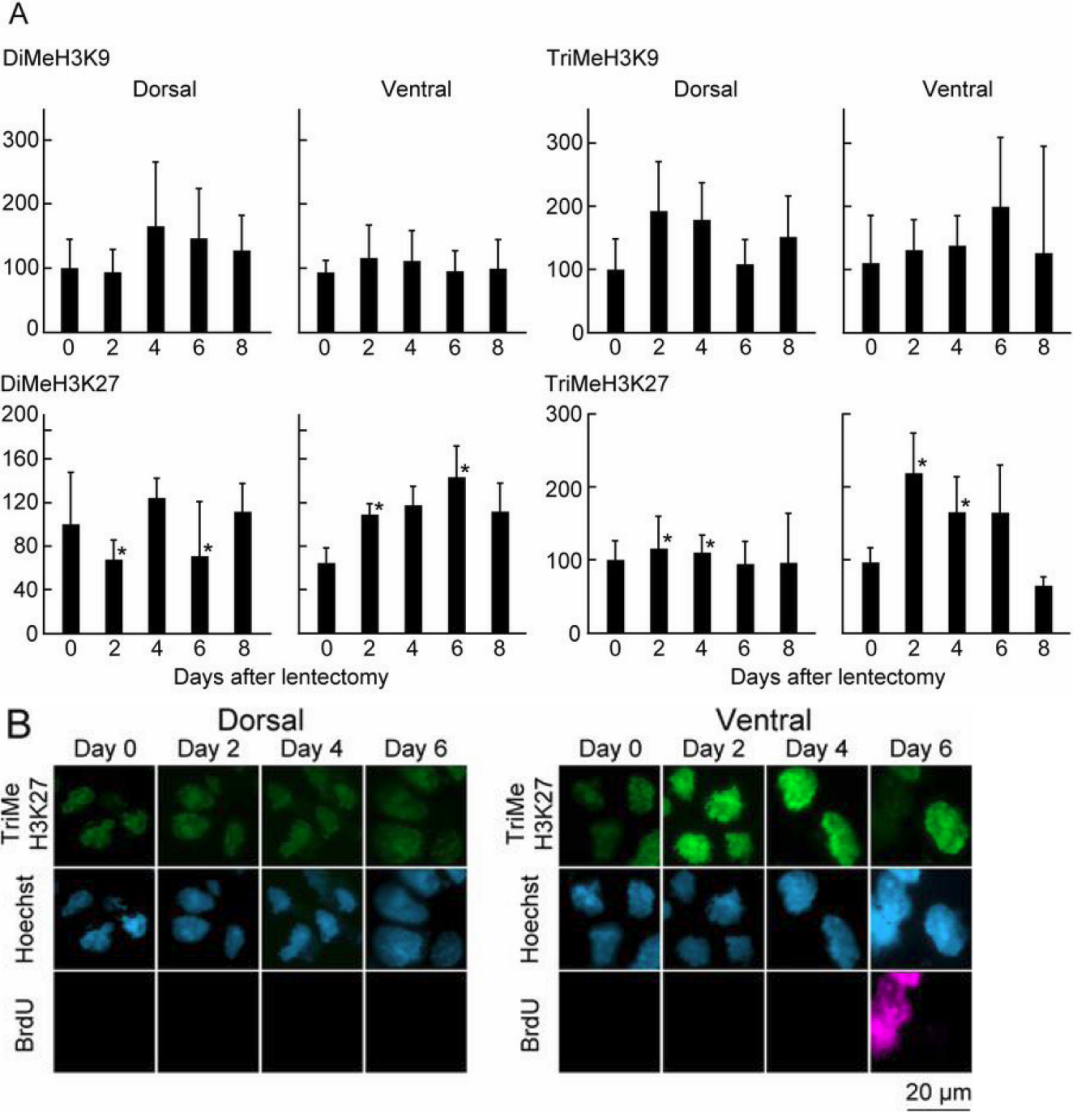
Changes in histone modifications related to gene repression during early lens regeneration. Same procedure as in Figure 1. **A**: Quantification of detected signal by immunohistochemistry using histone modification antibodies. **B**: Immunohistochemistry using TriMeH3K27 antibody, showing patterns in dorsal and ventral iris at different time points.

To examine the cells before cell cycle reentry, BrdU was administered every day starting the day before lentectomy. Eye balls were collected at different times thereafter and double-colored immunohistochemistry using BrdU and histone modification antibodies was performed. In this experiment signal intensities of histone modification were measured against PECs that did not incorporate BrdU. Signal intensities of each histone modification were normalized with that of Hoechst 33258 (Figure 1 and Figure 2). In these figures the mean value of normalized signal in dorsal iris on day 0 is represented as 100%.

Figure 1 shows changes in histone modifications related to gene activation [8,9]. After lentectomy global TriMeH3K4 and AcH4 (K5, 8, 12, 16) were increased in both of dorsal and ventral iris. In contrast AcH3K9 was high level on day 0 and decreased gradually by day 8. This indicates that each histone modification related to gene activation is differentially regulated during dedifferention of PEC. Such a coordination of decreasing of AcH3K9 and increasing of TriMeH3K4 and AcH4 could be a hallmark of chromatin regulation during newt dedifferentiation. This could mean that TriMeH3K4 and AcH4 modifications activate genes related to dedifferentiation and cell cycle re-entry. AcH3K9 is decreased during dedifferentiation meaning that it is probably involved in maintaining transcription of genes related to the differentiated state of intact iris. No modification showing consistency during the time period that we examined exhibited a clear dorsal/ventral difference.

**Figure 3:**
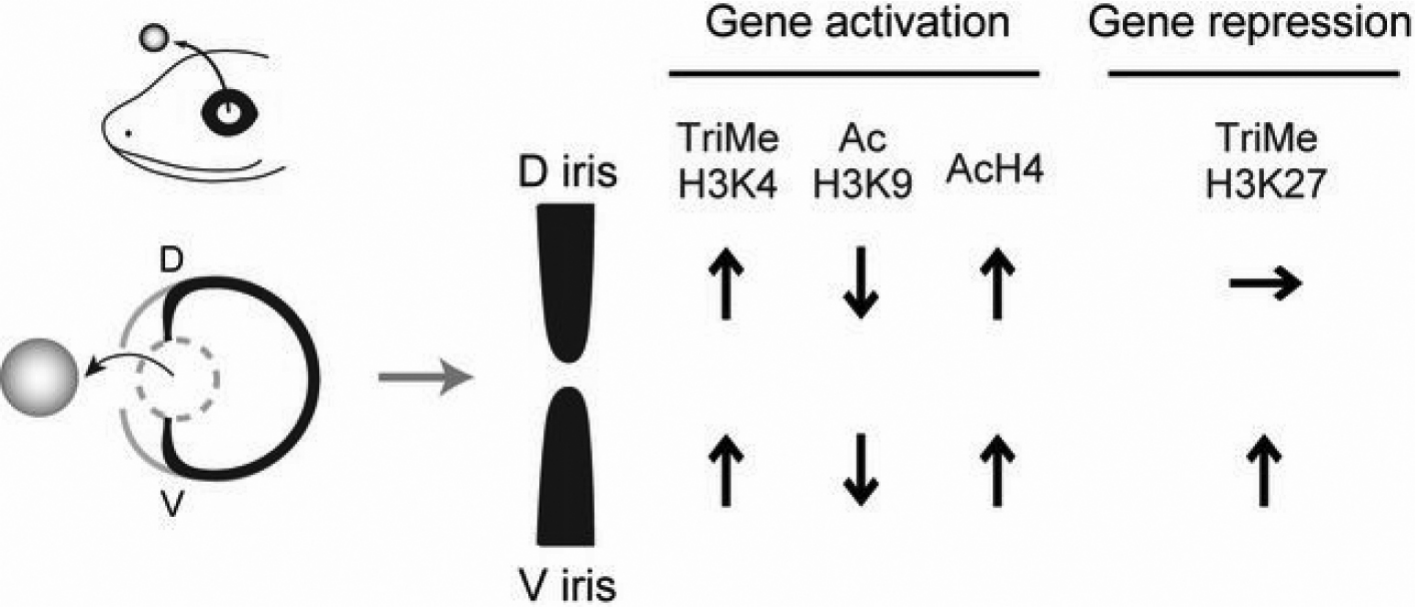
Summary of changes in histone modifications during dedifferentiation in lens regeneration. Only modifications, which are changed during dedifferentiation in relation to intact iris or to dorsal/ventral iris are indicated. D, dorsal iris; V, ventral iris.

Changes in histone modifications related to gene repression are shown in Figure 2. After lens removal the level of DiMeH3K9 and TriMeH3K9 were almost constant in both irises. Thus, we believe that these modifications do not play any significant role in regulating dedifferentiation. However, a dorso-ventral difference was found in TriMeH3K27. Although levels were not much changed in dorsal iris, they increased in ventral iris. Given the fact that this modification cooperates with polycomb group proteins and functions in gene silencing during development [10], this strongly suggests a correlation with inhibition of lens regeneration from the ventral iris. Another modification, DiMeH3K27, showed increased levels in the ventral iris at day 2 and 6 after lentectomy, but the values in the dorsal iris during dedifferentiation were not higher than the ones in the intact dorsal iris. Thus, this modification might not be significant for the dedifferentiation process. Figure 3 summarizes regulation of histone modifications during dedifferentiation.

A combination of different modifications, related to activation and repression of gene expression, seems to be crucial. In ES cells a similar regulation called bivalent histone modifications has been reported [11-14]. A vast majority of genes modified with TriMeH3K27 are co-modified with TriMeH3K4 in ES cells and the co-modified fraction is enriched in genes that function in development. The bivalent histone modifications are thought to poise genes for later activation while keep them inactivated. Recently it has been reported that in intact zebrafish silenced developmental regulatory genes contain bivalent TriMeH3K4 and TriMeH3K27 modiﬁcations and the silenced genes are converted to an active state by loss of TriMeH3K27 modiﬁcation during fin regeneration [15]. However, loss of TriMeH3K27 does not occur in newt dedifferentiation (Figure 2 and Figure 3). Rather, it is suggested that TriMeH3K27 exerts a dorso-ventral selectivity of lens formation by its increase in ventral iris. The data presented here point to global modifications and thus do not single out a particular molecular mechanism or pathway. However, the enzymes that mediate such modifications are known [16]. Thus, in the future it will possible to address in more specific ways the genetic pathways underlying the spectacular event of lens regeneration.
